# Local Infiltration Analgesia with Ropivacaine Improves Postoperative Pain Control in Ankle Fracture Patients: A Retrospective Cohort Study

**DOI:** 10.1155/2020/8542849

**Published:** 2020-03-09

**Authors:** Bao-Liang Li, Xizhe Liu, Lihua Cui, Wenqian Zhang, Hui Pang, Mingshan Wang, Hai-Qiang Wang

**Affiliations:** ^1^Department of Orthopaedic, The Seventh Affiliated Hospital, Sun Yat-sen University, Shenzhen 518107, China; ^2^Department of Spine Surgery, The First Affiliated Hospital, Sun Yat-sen University, Guangzhou 510000, China; ^3^Department of Orthopaedic, Binzhou People's Hospital, Binzhou 256600, Shandong Province, China; ^4^Institute of Integrative Medicine, Shaanxi University of Chinese Medicine, Xi'an 712046, Shaanxi Province, China

## Abstract

**Purpose:**

The study aimed at investigating the effect of local infiltration analgesia (LIA) with ropivacaine on postoperative analgesia for patients undergoing ankle fracture surgery.

**Methods:**

Consecutive patients were retrospectively included and analysed according to their medical records from July 2014 to August 2018 in a tertiary hospital. Inclusion criteria were patients undergoing open reduction and internal fixation (ORIF) for ankle fractures under general anaesthesia. Moreover, patients should have received intravenous patient-controlled analgesia (iPCA) or LIA + iPCA for postoperative pain relief. The primary outcome indicator was visual analogue scale (VAS) from 8 hours to 48 hours after surgery. Secondary outcomes included postoperative opioid requirement, need for rescue medication, opioid-related adverse effects, and wound complications.

**Results:**

In total, 89 consecutive patients were included in the study. There were 48 males and 41 females. The average age was 44.6 ± 7.0 years, and VAS scores were significantly lower in the LIA + iPCA group at 8 hours after surgery (1.51 ± 0.58 cm vs 4.77 ± 1.83 cm, *p* < 0.001). The time to first tramadol consumption was longer (580 ± 60.9 minutes vs 281 ± 86.4 minutes, *p* < 0.001). The time to first tramadol consumption was longer (580 ± 60.9 minutes vs 281 ± 86.4 minutes, *p* < 0.001). The time to first tramadol consumption was longer (580 ± 60.9 minutes vs 281 ± 86.4 minutes, *p* < 0.001). The time to first tramadol consumption was longer (580 ± 60.9 minutes vs 281 ± 86.4 minutes, *p* < 0.001). The time to first tramadol consumption was longer (580 ± 60.9 minutes vs 281 ± 86.4 minutes,

**Conclusions:**

The retrospective cohort study indicates that LIA with ropivacaine can provide better early postoperative pain management with a reduction of VAS scores for ankle fracture surgery. Patients receiving wound infiltration also experience decreased opioid consumption, a lower rate of analgesia-related side effects, and comparable wound complication rate.

## 1. Introduction

Severe postoperative pain has multiple detrimental effects on patients' recovery and quality of life. Patients with poorly managed pain are more prone to experience delay in physical activity and discharge after surgery, interfering with the participation in rehabilitation programs, eventually leading to unsatisfactory outcomes [1]. Updated management of postoperative pain mainly consists of iPCA. Compared with conventional intramuscular injections, iPCA improves postoperative pain relief and potentially reduces the hospital stay by enhancing patients' restoration [2]. Opioids are the most commonly used iPCA drug, with high effectiveness for alleviating moderate-to-severe postoperative pain without ceiling effects [3]. However, these drugs may lead to numerous opioid-related adverse effects, such as pruritus, vomiting, and nausea [3, 4].

Whereas iPCA remains the most commonly used technique for relieving postoperative pain, there has been an increased utilization of local infiltration analgesia (LIA) at the surgical site in the past decade [5]. The resurgence of LIA can be attributed to the availability of long-duration anaesthetics (e.g., ropivacaine and liposome bupivacaine). LIA has been reported to improve the quality of analgesia, decrease the morphine consumption, and reduce hospital stay [6]. However, the efficiency of this technique for postoperative pain relief is not always excellent. Miu al. reported that surgical site infiltration with ropivacaine did not significantly reduce pain or opioid consumption after thyroid surgery [7]. It is believed that the efficacy of local anaesthetics is highly correlated with surgical procedures [8]. Despite the redundancy of studies exploring the efficacy of LIA in various types of surgical procedures, few studies have addressed the use of local anaesthetics in ankle fracture surgery to date.

Therefore, we designed this retrospective cohort study aiming at evaluating the efficacy of LIA and iPCA in patients undergoing isolated ORIF of ankle fractures. The primary outcome was the VAS up to day 2 after surgery. Secondary outcomes included postoperative opioid requirement, need for rescue medication, opioid-related side effects, and wound complications. We hypothesized that the addition of LIA with ropivacaine after ankle fracture surgery would result in adequate pain control, significant decrease in opioid consumption, a lower rate of analgesia-related side effects, and comparable wound complication rate compared iPCA alone.

## 2. Materials and Methods

### 2.1. Study Design and Setting

This study was approved by the Institutional Ethics Review Board of Binzhou People's Hospital. We retrospectively reviewed the medical records of all patients who underwent ORIF of ankle fractures at Binzhou People's Hospital between July 2014 and August 2018. We choose this time period because LIA became widely adopted in our hospital as an adjunct to postoperative pain control since 2016. As well, surgical techniques, anaesthesia procedures, and other pain management protocols were unchanged during the study period. Eligible subjects were patients undergoing ankle fracture surgery with general anaesthesia alone at our centre. Exclusion criteria were pathologic or paediatric fracture, open fracture, history of chronic pain, and patients with more than one part of injuries to the body. To eliminate confounding bias related to surgical types, we excluded patients treated only with isolated percutaneous screw fixation. The ankle fractures were classified into three types according to the location of fractures: lateral malleolar fractures, bimalleolar fractures, and trimalleolar fractures. Clinical databases were reviewed to determine demographic characteristics, including age, gender, fracture type, surgery time, and incision length.

### 2.2. Surgical Techniques

All surgeries were carried out between 7 to 14 days after admission to decrease the incidence of soft tissue complications. Surgical procedures were performed under general anaesthesia. All surgeries were performed by senior Orthopaedic surgeons. The ankle fractures were treated via different operative approaches (e.g., medial approach, lateral approach, posterolateral approach, or modified anteromedial approach) depending on fracture types and the location of major fragments. Fractures were fixed in a standard manner, with screw-plate system, cannulated screws, and tension band wiring.

### 2.3. Aftercare

Included patients were divided into two groups according to the regimens of postoperative pain relief: either LIA + iPCA or iPCA. The LIA was with ropivacaine (0.5% ropivacaine, total amount was between 15 ml and 30 ml depending on length of surgical incision) injected into the dermis and subcutaneous tissue surrounding the incision. The procedure was conducted by the surgeon before incision was sutured. All patients received the same iPCA pain control regimens. An intravenous patient-controlled analgesia pump was connected to patients postoperatively. The pump contained morphine with a bolus of 0.5 ml and a lockout interval of 10 minutes.

Since the Orthopaedic ward adopts a pain management mode, the nurses routinely evaluate the patient's postoperative pain intensity by a 10-cm horizontal visual analogue scale (VAS, 0 cm = no pain, 10 cm = maximum pain) every eight hours for two days. The VAS is a numeric rating scale tool for assessing pain intensity in which 0 indicates no pain at all and 10 indicates worst pain imaginable. If patients complained of poor pain management despite the use of iPCA, they were administered 50 mg of intramuscular tramadol as rescue medication.

The total dose of morphine administered via iPCA was examined when the pump was removed 2 days after surgery.

Further information concerning opioid-related adverse effects (nausea and vomiting) was collected from patients' medication records. Any complications relating to wound were also recorded.

### 2.4. Outcome Measures

The primary endpoint variable was the visual analogue scale (VAS) from 8 hours to 48 hours after surgery. Secondary outcome variables included postoperative opioid requirement (morphine via iPCA) and need for rescue medication in the 48 hours after surgery, opioid-related side effects, and wound complications.

### 2.5. Sample Size

Postoperative VAS score was used to calculate the least sample size. On the basis of previous researches, we used a minimal clinical significant change in VAS score of 1.8 cm [9] and a standard deviation of 1.69 cm [10, 11]. With an alpha level of 0.05, a power of 90%, and an anticipated dropout rate of 20%, the least needed sample size per group was 23 patients.

### 2.6. Statistical Analysis

IBM SPSS Statistics version 20.0 (IBM Corporation, Armonk, NY, USA) was used to perform statistical analysis. Means and standard deviations (SD) were used to evaluate the continuous variables. The Kolmogorov–Smirnov normality test was used to test whether continuous variables were normally distributed. Depending on the results of the Kolmogorov–Smirnov normality test analysis, either Student's *t*-test or Mann–Whitney *U* tests were performed. Categorical data and frequencies were analysed by Pearson's chi-square test or Fisher exact tests where appropriate. A *p* value less than 0.05 was considered statistically significant.

## 3. Results

Between July 2014 and August 2018, 103 patients underwent ORIF of ankle fractures at our hospital. A total of 89 patients were available for the final analysis of our study. 39 patients (44%) received iPCA alone, 50 patients (54%) received LIA and iPCA. There were no significant differences between the two study groups with respect to age, gender, fracture type, surgery time, and length of surgical incision (Table 1).

**Figure 1:**
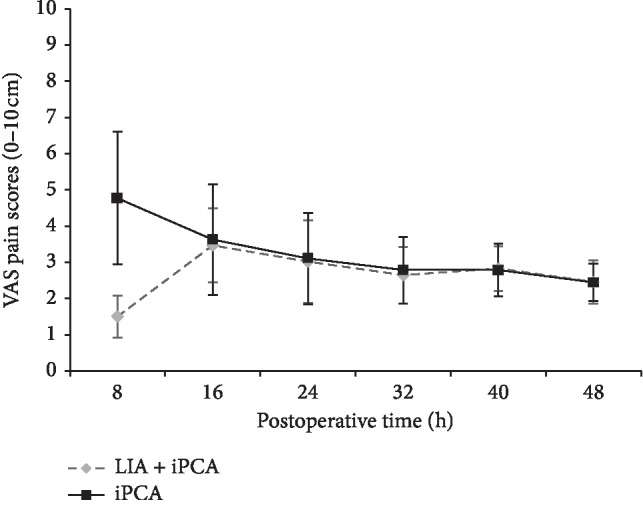
The pain scores (means and SD) after ORIF of ankle fractures, as rated on 10-cm VAS. The LIA + iPCA group had significantly lower VAS score 8 hours after surgery (*p* < 0.001).

In terms of postoperative VAS score, the LIA + iPCA group had significantly lower score (1.51 cm ± 0.58 cm) at 8 hours compared with the iPCA group (4.77 cm ± 0.83 cm) (*p* < 0.001). There was no significant difference between the two treatment groups regarding the VAS score since 16 hours after surgery (Table 2) (Figure 1).

**Figure 2:**
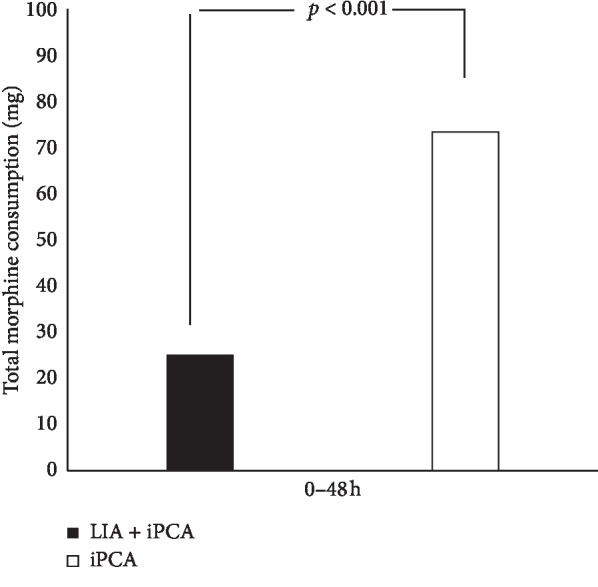
Mean (SD) total morphine consumption in the 48 hours after surgery.

As for postoperative morphine requirement in the 48 hours after surgery, the mean (±SD) total morphine consumption was 25.1 (±6.3) mg for the LIA + iPCA group compared to 73.4 (±8.2) mg for the iPCA group, and this was a significant difference (*p* < 0.001) (Figure 2).

**Figure 3:**
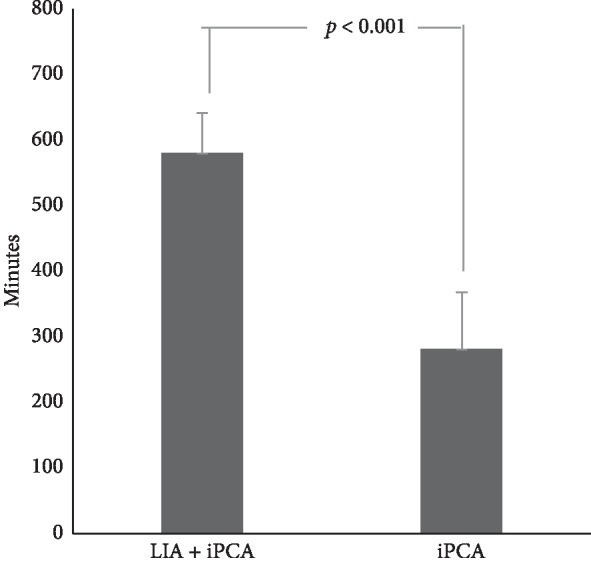
The time to first tramadol consumption after surgery (*p* < 0.001).

**Figure 4:**
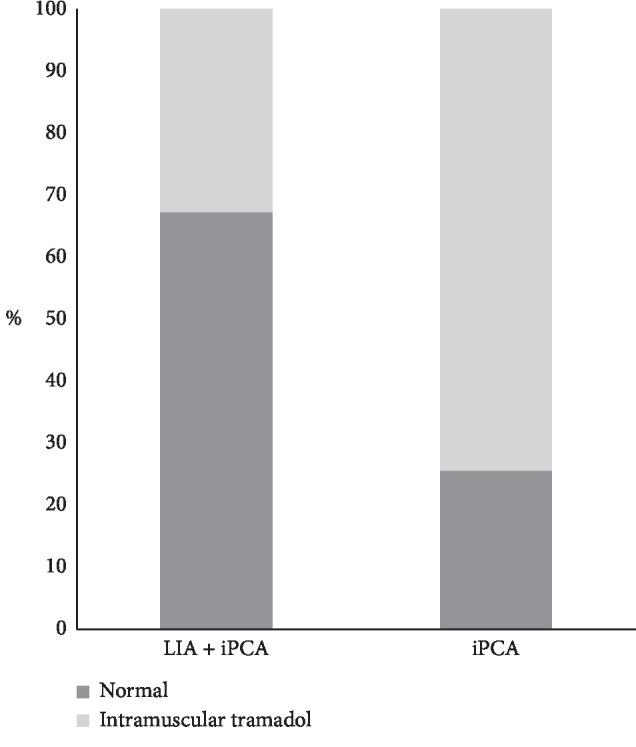
The percentage of patients who need intramuscular tramadol as rescue medication in the first 48 hours after surgery (*p*=0.04).

The time to first tramadol consumption was 580 ± 60.9 minutes in the LIA + iPCA group compared to 281 ± 86.4 minutes in the iPCA group (*p* < 0.001) (Figure 3). There was a significant difference between groups on the number of patients who need intramuscular tramadol as rescue medication in the first 48 hours after surgery (18 vs 26 for the LIA + iPCA and iPCA groups, respectively, 36% vs 67%, *p*=0.04) (Figure 4).

**Figure 5:**
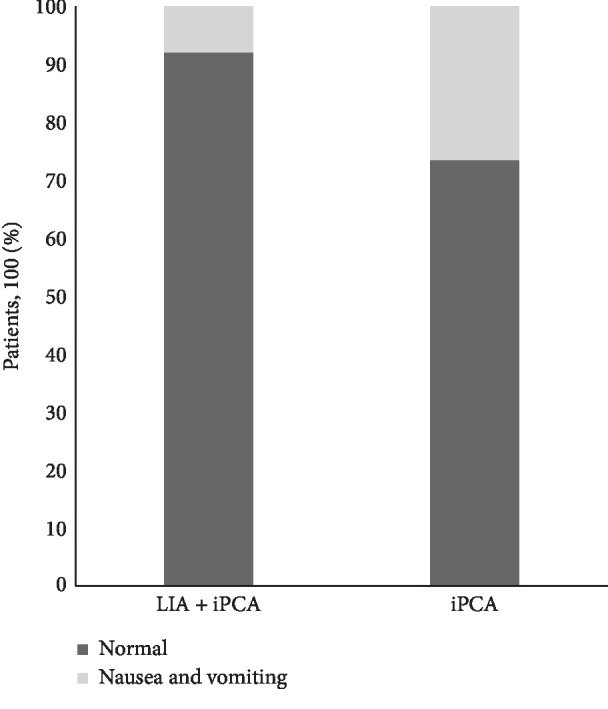
Percentage of nausea and vomiting patients in the 48 hours after surgery (*p*=0.023).

We noted a significant reduced incidence of postoperative nausea and vomiting (*p*= 0.023) in the LIA + iPCA group (8%) compared with patients in the iPCA group (26.6%) (Figure 5).

**Figure 6:**
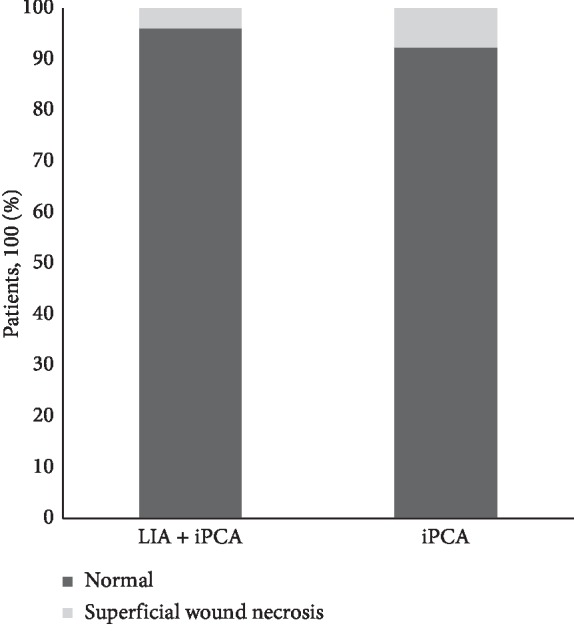
Percentage of superficial wound necrosis patients after surgery (*p*=0.45).

There were 5 cases of superficial wound necrosis noted after surgery, 2 (4%) in group LIA + iPCA and 3 (7.7%) in group iPCA (*p*=0.45) (Figure 6). All cases were cured by local wound care without antibiotic application. No major wound complications occurred, and none of the patients needed reoperations for wound complications in both groups during the inhospital stay. We found no adverse drug related events of ropivacaine in the LIA + iPCA group.

## 4. Discussion

Poorly managed postoperative pain after ankle fracture fixation surgery not only leads to an increase in hospital stay and a decrease in life quality but also elicits worse functional outcome [12–14]. Tremendous efforts have been made on controlling postoperative pain, such as opioid therapy and multimodal analgesic techniques. Amongst these treatment modalities, peripheral nerve block has gained popularity as an adjunct to postoperative pain control in ankle fracture surgery. Blumenthal et al. found that in major ankle surgery operations, a combination of continuous popliteal and femoral nerve block significantly relieved postoperative pain and postoperative morphine consumption [15]. In a systematic review of regional anaesthesia for foot and ankle surgery, Pearce et al. reported that the peripheral nerve block was associated with high levels of patient satisfaction and substantial reduction in hospital costs [16]. Although the peripheral nerve block is effective, technical difficulties related to the placement of catheters and potential complications (such as nerve injury and systemic toxicity) have led to its routine use restricted to a limited number of institutions [3, 17]. Studies have demonstrated that the use of peripheral nerve block to improve postoperative analgesia is very limited worldwide [18, 19].

In recent years, LIA has been increasingly used for postoperative pain relief in a variety of surgical procedures, including abdominal, cardiothoracic, and orthopaedic. The reported advantages of this technique include favourable safety profile, reduced morphine consumption, improved pain control, and simplified technical procedures. Studies have shown that LIA may be an effective alternative to the peripheral nerve block in pain relief Lefevre et al. conducted a prospective cohort study to compare the efficacy of postoperative analgesia with femoral nerve block and LIA in patients undergoing ligament reconstruction surgery [20]. They found that the femoral nerve block is less effective than LIA on early postoperative pain relief. Several studies have shown that LIA results in better pain control, superior knee function, and more rapid discharge from hospital, comparing with peripheral nerve block techniques of pain relief for both THA and TKA [21–23].

However, the efficacy of local anaesthetics is highly correlated with surgical procedures and local anaesthetic agents. Miu et al. reported that surgical site infiltration with ropivacaine did not significantly reduce pain or opioid consumption after thyroid surgery [6]. To our knowledge, few studies to date have explored the efficacy of LIA with ropivacaine after ankle fracture surgeries.

Ropivacaine is a new amino-amide local anaesthetic agent introduced into clinical use in the early 1990s [24]. It has been advocated as a preferred local anaesthetic for LIA by many investigators [25, 26]. Due to the vasoconstrictive properties of ropivacaine capable of decreasing systemic absorption, it was considered to produce a long-lasting local anaesthetic block that effectively manages postoperative pain [27, 28]. Ropivacaine shows a more favourable clinical safety profile than bupivacaine, with decreased cardiotoxicity and central nervous system toxicity [29–31]. For local infiltration, the recommended dose of ropivacaine should be no more than 200 mg, with a volume less than 100 ml. The does used in this study is 75 to 150 mg. We observed no signs of local or systemic toxic reactions, supporting the administration of bupivacaine.

In this retrospective cohort study, we investigated the postoperative analgesia efficiency of LIA with ropivacaine in ankle fracture surgery. Our results demonstrated that LIA with ropivacaine in ankle fracture surgery was associated with dramatic reduction of early postoperative pain, less opioid consumption, decreased number of patients who need rescue medication, and longer time to first rescue medication after the surgery.

The significant reduction of early postoperative pain scores, less postoperative opioid consumption, and less rescue tramadol administration in the LIA + iPCA group confirms the better postoperative pain control provided by local infiltration analgesia in ankle fracture surgery. Our results are consistent with the reports of Kalogera et al. who reported a significant reduction in patient-controlled analgesia use and rescue medicine requirement after LIA [32]. However, we failed to show any significant reduction in VAS scores with the use of local infiltration analgesia after 8 hours post-surgery. We believed that this could be due to the fact that LIA mainly reduces the immediate postoperative pain. Beatrice et al demonstrated that LIA lower visual analogue scale scores at 1, 3, and 6 hours after surgery, while less morphine was needed even at 12 hours after surgery [4].

There are a number of limitations existing in our study. First, due to the retrospective and nonrandomized design of this study, our results may have been affected by confounding or unrecognized variables. Second, we were unable to record the opioid consumption at every time point that VAS scores were evaluated. Therefore, the LIA action duration was unclear. Additionally, lack of long-term clinical evaluation, such as an assessment of whether LIA is associated with less chronic pain, was conducted in our study. Nevertheless, our retrospective cohort study presents beneficial line of evidence on the efficiency of LIA in the pain management of ankle fractures.

## 5. Conclusions

Local infiltration analgesia is a safe and valuable postoperative pain management technique in patients undergoing ankle fracture fixation surgery. Patients who receive wound infiltration can experience a significant decrease in early postoperative pain, reduction in opioid consumption, a low rate of analgesia-related adverse effects, and comparable wound complication rate.

## Figures and Tables

**Table 1:** Demographic data.

Variable	LIA + iPCA (*n* = 50)	iPCA (*n* = 39)	*p* value
Age (y)^∗^	44.1 ± 6.8	45.3 ± 7.3	0.426
Gender (M/F)^†^	28/22	20/19	0.658
Surgery time (min)^∗^	101.7 ± 25.1	95.4 ± 20.9	0.210
Incision length(cm)^∗^	15.3 ± 7.2	16.7 ± 5.8	0.325
Number of fractures			
Lateral malleolar fractures^†^	7	3	0.437
Bimalleolar fractures^†^	28	20	
Trimalleolar fractures^†^	15	16	

^∗^Values are expressed as means ± SD. ^†^Values are given as the number of patients. *p* values based on the independent *t*-test or Pearson chi-square test.

**Table 2:** Postoperative VAS scores (cm).

Variable	LIA + iPCA	iPCA	*p* value
VAS 8 h	1.51 ± 0.58	4.77 ± 1.83	<0.001
VAS 16 h	3.47 ± 1.02	3.63 ± 1.53	0.556
VAS 24 h	3.02 ± 1.14	3.11 ± 1.26	0.725
VAS 32 h	2.65 ± 0.78	2.79 ± 0.92	0.440
VAS 40 h	2.83 ± 0.61	2.79 ± 0.72	0.777
VAS 48 h	2.46 ± 0.59	2.45 ± 0.51	0.933

Values are expressed as means ± SD. *p* values based on the independent *t*-test.

## Data Availability

The datasets used to support the current study are available from the authors upon reasonable request.
